# Acute Promyelocytic Leukemia: an Experience on 95 Greek Patients Treated in the All-Trans-Retinoic Acid Era

**DOI:** 10.4084/MJHID.2011.053

**Published:** 2011-11-28

**Authors:** Maria Pagoni, Maria Garofalaki, Fotios Panitsas, Kalliopi Manola, Katerina Psarra, Panagiotis Economopoulos, Aggeliki Vourtsi, Marios Antoniades, Kostas Gkirkas, Evangelia Tzouvara, Fotis Katis, Chrystalla Prokopiou, Irene Tziotziou, Artemis Balta, Eleni Lemissiou, Panagiotis Tsirigotis, Panagiotis Repoussis, Nicolas Harhalakis

**Affiliations:** 1 Hematology-Lymphoma Department - BMT Unit, Evangelismos Hospital, Athens, Greece; 2 Department of Cytogenetics, Laboratory of Health Physics and Environmental Hygiene, National Center for Scientific Research (NCSR) “Demokritos”, Athens, Greece; 3 Immunology and Histocompatibility Department, Evangelismos Hospital, Athens, Greece; 4 Haematology Clinic, Nicosia General Hospital, Cyprus; 5 Attikon Hospital Athens, Greece; 6 Hematology Department, University Hospital of Patras, Greece; 7 Metaxa Anticancer Hospital, Peiraias, Greece; 8 Haematology Clinic, Limassol General Hospital, Cyprus

## Abstract

Acute promyelocytic leukemia (APL) is highly curable with the combination of all-transretinoic acid (ATRA) and anthracycline based chemotherapy, but the percentage of early deaths remains high. In the present study, we report the clinical, immunophenotypic, cytogenetic and molecular characteristics and outcome of APL patients diagnosed and treated in various Hospitals of Greece and Cyprus.

We describe the data of ninety-five APL patients who were diagnosed during the last 15 years. Seven (7.4%) newly diagnosed APL patients died due to intracranial hemorrhage within 72 hours of presentation. All but two patients were induced with ATRA alone or ATRA plus chemotherapy. The early death rate was 14.9%. After induction all 80 evaluable patients achieved complete hematologic remission. The cumulative incidence of relapse was 18.3%. Eight of the ten relapsed patients were successfully salvaged, while both patients with molecularly resistant disease died during salvage treatment. Overall survival (OS) at 5 years was 78.4% and disease free survival (DFS) 73.6%. In multivariate analysis of OS age over 60 years, DIC at diagnosis and marginally major hemorrhage at presentation were identified as adverse prognostic factors. In the subgroup of patients with available data on FLT3 mutation status (49 out of 94), ITD positivity also remained as an independent prognostic factor in the final model of OS, together with major hemorrhage and marginally high Sanz score. We found a close correlation between the CD2 expression and the development of the differentiation syndrome (DS). In conclusion, the main problem in managing patients with APL is still the high early death rate.

## Introduction

Acute promyelocytic leukemia (APL) is a distinct subtype of acute myelogenous leukemia (AML) with special biological, morphological, cytogenetic, and molecular characteristics and also clinical features. Morphologically APL is identified by the FAB classification as M3 subtype (hypergranular) and its microgranular variant (M3v).^1^–^5^ Cytogenetically is characterized by the t(15;17)(q22;q21) which is considered to be a favorable cytogenetic aberration.^6^–^9^

In rare cases other translocations as t(11;17), t(5;17) can be detected.^10^ Additional chromosome aberrations to t(15;17) have been observed in 23%–43% of APL cases without changing the favorable prognosis.^11^–^15^

At the molecular level, the result of the t(15;17) is the formation of two functional fusion genes, *PML-RARα* and *RARα-PML* on the derivative chromosomes 15 and 17 respectively.^5^,^8^,^9^,^16^ A variety of *PML-RARα* transcripts [bcr1 or Long (L), bcr3 or Short (S), bcr2 or Variable (V)] are produced due to different breakpoints in *PML* gene and alternative splicing in chromosome 15.^17^,^18^ The *FLT3* gene aberrations, including internal tandem duplications (ITDs) and D835 (tyrosine kinase domain, TKD) mutations occur in 30%–50% in APL. However, the significance of FLT3 mutations as a prognostic factor is not firmly established.^19^–^22^

With current treatment strategies with all-trans-retinoic acid (ATRA) in combination with anthracycline based chemotherapy, approximately 70%–80% of patients with newly diagnosed APL carrying PML/RARα achieve long-term remission and are probably cured. However, relapses occur in 10%–30% of patients. The role of cytosine arabinoside and other agents remains controversial. For patients in whom chemotherapy is contraindicated, for elderly patients and for some relapsing patients Arsenic Trioxide (ATO) is a suitable alternative, as a single agent or in combination with ATRA. There is not agreement on the most appropriate consolidation therapy but generally two to three cycles of anthracycline-based chemotherapy are considered mandatory. Finally, there is an open debate about the need for maintenance treatment.^23^–^31^

Besides the excellent results, serious problems still remain: a high early death rate (up to 10% for patients who enrolled in clinical trials and higher in real life), cardiotoxicity, secondary myelodysplastic syndromes, and relapse rising up to 20%–30% in high risk patients.^30^,^32^,^33^–^37^

The APL published data in Greek patients are very limited, usually restricted to case reports or small series.^38^–^41^

In the present study, we report on 95 patients from Greece and Cyprus in order to clarify the clinical, cytogenetic and molecular features as well as their outcome.

## Design and methods

Between January 1996 and June 2011 a total of 95 patients with APL were diagnosed and treated in 6 tertiary hospitals in Athens, Patras, Nicosia and Limassol. The diagnosis was confirmed with cytogenetics for the presence of t(15;17) and/or molecular studies for the presence of the PML/RARα fusion gene. Informed consent was obtained from all patients according the Declaration of Helsinki and all the protocols were approved by the Research Ethics Committee of the participating hospitals.

Laboratory monitoring at diagnosis consisted of complete blood count, coagulation studies, and biochemical profile. Bone marrow aspirates were collected for morphology, immunophenotyping, molecular studies and cytogenetics. A comprehensive cardiac assessment was performed. The disease status was assessed with morphology and molecular studies in bone marrow aspirates in all patients before each cycle of chemotherapy, every three months during the first year after the treatment was completed, every four months during the second year, and subsequently every six months up to five years. In case of a PCR positive result after the third consolidation course or during subsequent follow-up a new sample, collected at least 2 weeks but no more than a month apart, was obtained. In case of repeated PCR positivity the patient was defined as having molecular relapse.^27^,^28^,^31^ The Sanz risk score was used in order to decide the treatment intensity in some patients and for analyzing the results.^42^

### Treatment protocols

During the first period (up to 2004) most patients followed the AIDA0493 protocol, as it has been described previously.^43^ In brief, induction: idarubicin+ATRA, 1^st^ consolidation: idarubicin+cytarabine, 2^nd^ consolidation: mitoxantrone+etoposide, 3^rd^ consolidation: idarubicin+cytarabine+6-thioguanine. From 2004 onwards most patients were treated according to the PETHEMA protocols (LPA1999 and LPA2005), as they have been described previously.^35^,^44^,^45^ In brief, induction: idarubicin+ATRA, 1^st^ consolidation: idarubicin, 2^nd^ consolidation: mitoxantrone, 3^rd^ consolidation: idarubicin. ATRA was added in each consolidation cycle in the intermediate and high risk patients (protocol LPA1999). In protocol LPA2005 ATRA was added in consolidation cycles of all patients, irrespective of their risk group. In addition, cytarabine was added in the 1^st^ and the 3^rd^ cycle of consolidation in the high risk patients. A few patients were treated according to the European APL93 and APL2000 protocols.^30^,^46^–^48^ In brief, induction: ATRA+daunorubicin+cytarabine, 1^st^ and 2^nd^ consolidation: daunorubicin+cytarabine. In most patients, regardless the treatment protocol, maintenance treatment with ATRA+mercaptopourine+methotrexate (ATRA+6-MP+MTX) or ATRA alone was given for two years.^28^,^49^–^51^ No CNS prophylaxis was given.^27^,^52^

### Supportive care

When coagulopathy was present fresh-frozen plasma and platelet transfusions were given with a platelet target of 30×10^9^/L. Heparin or tranexamic acid was not given. After resolution of the coagulopathy fresh-frozen plasma was not given and the platelet transfusions were given when hemorrhagic manifestations and/or infection were present.^27^,^28^ Prophylaxis for the retinoic acid syndrome was not given, as the effectiveness of this procedure is not established according to previous reports.^47^,^53^–^57^

The prophylaxis during neutropenia and the management of neutropenic fever or infection followed the protocols of the participating hospitals.

### Immunophenotypic studies

Immunophenotypic studies were performed on erythrocyte lysed whole bone marrow samples with directly conjugated monoclonal antibodies. The expression of HLA-DR, CD34, CD15, CD13, CD33, MPO as well as the side scatter (SS) of the abnormal cell population was assessed. Various lymphoid markers (including CD2 and CD56) were also used in order to assess their aberrant expression.^27^,^58^–^61^

### Cytogenetic studies

Chromosome studies were performed on unstimulated bone marrow cells, cultured for 24 and 48 hours at the time of diagnosis, after the end of treatment and when any suspicion for the development of myelodysplastic syndrome was raised.^27^ Cytogenetic analyses were performed on trypsin G-banded chromosome preparations and imaging and karyotyping were performed via microscopy and computer imaging techniques. Karyotypes were described according to the International System for Human Cytogenetic Nomenclature (ISCN) 2009. Whenever possible at least 20 metaphases were analyzed in each case.

### Molecular studies

Conventional nested reverse transcription PCR (RT-PCR) was used for the detection of the various isoforms of the *PML-RARα* hybrid gene.^5^,^17^,^27^,^62^ The same method was used for minimal residual disease (MRD) monitoring until early 2004. Subsequently, for a more accurate MRD assessment we optimized the RQ-PCR protocols of the three *PML-RARα* isoforms for use with the Lightcycler®/Roche and we established a standard approach of fluorescence data acquisition. MRD assessment was always performed in bone marrow samples.^63^–^68^ DNA or RNA was used in PCR or RT-PCR respectively for the detection of the internal tandem duplication (ITD) or the point mutation D835 (TKD) of the *FLT3* gene.^19^,^69^

### Outcome definitions

Complete hematologic remission (CHR) and relapse were defined according to the National Cancer Institute criteria.^70^,^71^ Molecular remission was defined upon a negative PCR for the *PML-RARα* hybrid gene, as described above. Early death was defined as death occurring during induction therapy or during the aplasia that follows induction. Death before treatment was considered “early death” but was also reported separately. Molecular relapse was met when there were two positive PCR results for the *PML-RARα* hybrid gene, at least two weeks apart each other, in a patient being in molecular remission. The differentiation syndrome was defined as “definitely present” or “probable” according to Frankel et al.^27^,^72^,^73^

### Statistical methods

Nominal variables were summarized with frequencies and percentages, while continuous variables with median value and range. Comparisons between groups were done using the Mann-Whitney or Kruskal-Wallis test for continuous variables, and Pearson’s chi square or Fisher’s exact test for categorical data.

OS was calculated from the first day of induction treatment, while DFS was calculated from the date of complete hematologic response. Molecular and/or hematologic relapse, PML/RARα PCR positivity at the end of consolidation treatment, diagnosis of MDS and death irrespective of cause were defined as DFS events. OS and DFS were estimated by the Kaplan-Meier method.

Response and relapse cumulative incidences were calculated considering death as competing risk. Cumulative incidence of relapse (CIR) was calculated from the date of complete hematologic remission. Molecular or hematologic relapse and PCR positivity at the end of consolidation were defined as relapse events.

Effects on survival outcomes were tested by the log-rank test (OS and DFS) and Cox proportional hazards model. Cause specific hazard analysis was done in the presence of competing risks. Significant (p≤0.1) predictors from univariate analysis were further tested in multivariate models. The final model in multivariate analysis was reached by stepwise backward selection.

Logistic regression was employed for analysis of early death rate and differentiation syndrome risk.

All analyses were performed with STATA 11 software (StataCorp. 2009. *Stata Statistical Software: Release 11*. College Station, TX: StataCorp LP).

## Results

Demographic and baseline characteristics of the patients are shown in Table 1.

In nine patients the disease was therapy related (t-APL). Six patients had been treated with chemotherapy or irradiation because of some malignant disorder. In addition, there were three patients with multiple sclerosis that had been treated with mitoxantrone. Details for these patients are shown in Table 2.^74^–^78^

### Immunophenotyping

Most patients studied showed an “abnormal” cell population with intermediate to high side scatter in the CD45/SS dot plot. All patients but one (59/60, 98.3%) showed the characteristic absence to low percentage of HLA-DR. Actually this particular patient showed the variant translocation t(11;17). Only 3/58 (5.2%) were CD34 positive. CD15 positivity was not so rare, to be found in 12/62 patients (19.3%). The sharp strong expression of CD33 was a constant feature in all patients and it was accompanied by variable CD13 expression. MPO, where studied, was found positive in all patients. CD11b was found positive in a low percentage of patients (3/43, 7.0%) and CD117 expression was variable. Aberrant expression of lymphoid markers was found in 8/49 (16.3%) patients. CD2 expression was the more frequent finding (7/44, 15.9%), whereas CD7 was found in only one patient (1/60, 1.7%) and CD56 in two patients (2/49, 4.1%). Aberrant expression of lymphoid markers was not associated with bad prognosis (OS and DFS); as we observed only one molecular relapse event and no deaths among these eight patients. There was a close correlation between the expression of lymphoid markers and the development of the differentiation syndrome: 7/8 cases expressing lymphoid markers developed DS. No other correlation was found between immunophenotype and other features or outcomes of the patients, but it has to be stressed that the numbers were rather small.^58^–^61^,^79^

### Chromosomal abnormalities

Comprehensive karyotypic results were available in 68 patients. The detailed karyotypic results are shown in Table 3. Normal karyotype was found in 9 patients (13.2%).

Among the 59 cases with abnormal karyotype there were 42 cases with a single abnormality, seven cases with two abnormalities, and 10 cases with ≥3 abnormalities (complex karyotype). The t(15;17) was found in 52 cases (76.5%) and it was the sole abnormality in 37 cases. In 15 cases (28.8%) additional chromosomal abnormalities (ACAs) were present. Among the ACAs trisomy 8 was by far the most frequent;^12^–^15^ it was present in 7/15 cases. One patient showed t(8;21) in addition to t(15;17). He was treated with a hybrid protocol (AIDA and a conventional AML protocol) but he never achieved molecular CR.^80^,^81^ There was not any difference between the patients with ACAs and those without ACAs concerning the clinical features (data not shown) or the response to treatment.^11^,^15^

### Molecular findings

The presence of *PML-RARα* was tested in all but two patients and it was detected in 92/93 cases (98.9%). It was absent in the case with t(11;17), as expected. In 75 patients the PML-RARα isoforms were determined: L isoform (bcr1) in 38 cases (51%), V isoform (bcr2) in 5 cases (7%), and S isoform (bcr3) in 32 cases (42%).^5^,^17^,^18^ There was a striking difference in the frequency of the isoforms between the patients from Greece and those from Cyprus. In the Greek origin patients the L isoform was present in 36/64 cases (56.25%) and the S isoform in 24/64 (37.5%) while the corresponding figures for the Cyprus origin patients was 2/11 (18.2%) and 8/11 (72.7%) (p=0.036).

Fifty one bone marrow samples were analyzed for *FLT3* mutations at presentation. Mutations were found in 20 cases (39%): ITD in 11 cases and TKD in 9 cases.^19^,^20^,^82^ The presence of *FLT3* mutations was significantly associated with intermediate/high risk score (p=0.035).^82^ The microgranular variant was rather more frequent in *FLT3* mutated cases (p=0.09). Coagulopathy was significantly more prevalent among *FLT3* mutated cases (p=0.037). *FLT3* ITD was an adverse prognostic factor for early death and OS (p=0.017) but not for response rate or relapse incidence.^19^,^20^,^82^ In one patient we observed a constant positivity for *FLT3* TKD for 4 years while he remained in molecular remission.^19^,^21^

### Clinical course

At presentation laboratory findings of disseminated intravascular coagulation were present in 54/95 patients (57%). In 26 cases major hemorrhagic events were noticed. There were 9 patients with intracranial bleeding. Hemorrhage was the main cause of death in 9 patients: 3 within the first 24 hours, 3 at day two, and the others at day 8, 10, and 14. Two of these patients died before specific treatment was given. Twenty six patients presented with infection.^46^,^47^,^83^

### Induction therapy

Induction treatment was given in 88 patients: AIDA protocol (n=69),^43^ ATRA alone (n=4),^84^–^86^ ATRA+idarubicin+cytarabine (n=13).^30^ Two patients misdiagnosed as AML-M2 were treated with “3+7”.^87^ When the diagnosis was confirmed they followed the AIDA protocol. One patient discontinued treatment because cytogenetics showed t(11;17) and her age in conjunction with poor performance status prohibited further treatment.^88^,^89^ She died a few weeks later. She was excluded from further analysis. Twenty seven patients (31%) developed differentiation syndrome.^73^ In 5 cases (6%) this was definite. In all the patients developing DS, dexamethasone and furosemide were administered. ATRA was temporarily discontinued in 9 cases and its dose reduced in one case. In 3 cases the DS was the main or a contributory cause of death during induction. In all the other cases it resolved within a few days.^27^,^72^ In 4 patients (5%) pseudotumor cerebri developed. All these patients were female and their age was 19 (n=2), 22 and 67 years old. Temporal discontinuation or dose reduction of ATRA in conjunction with dexamethasone and diuretic administration was followed by resolution of the symptoms.^27^,^43^ Infection experienced 62 patients during induction with neutropenic fever being the most common manifestation (n=42). In one case, pneumonia was the direct cause of death and in two cases a contributing cause. Eight patients died during induction. Median time to death was 14 days (range, 8–55). All but one of those patients died before being evaluated for response. The patient that died on day 55 was in CHR. Causes of death: intracranial hemorrhage (n=3), pneumonia and differentiation syndrome (n=2), pneumonia (n=1) septic shock (n=1), and respiratory failure because of differentiation syndrome (n=1). All 80 patients that completed induction and survived the early period achieved CHR at a median of 35 days (range, 19–99). These patients were candidates for consolidation.

### Consolidation therapy

From the 80 patients that achieved complete hematologic remission after induction, 76 proceeded to consolidation. One patient died because of lung infection while in CHR. One patient 84 years old was induced with ATRA alone and achieved molecular remission.^84^–^86^ Because of her poor performance status she was offered no further treatment. She had a molecular relapse after 39 months and a hematologic relapse after 30 further months. She then received AIDA induction 43 but she died in aplasia. The other two patients decided by themselves to discontinue further treatment. The first consolidation cycle varied according to the protocol that the patient followed: AIDA (n=39),^43^ PETHEMA (n=24),^35^,^44^,^45^ European APL (n=7),^30^,^46^–^48^ other (n=6). There were two deaths because of infection and one patient experienced severe organ toxicity after the 1^st^ consolidation cycle and, therefore, 73 patients received the 2^nd^ consolidation cycle. The patients followed the same protocols. One patient died after the 2^nd^ consolidation because of infection, one developed severe organ toxicity, one relapsed, one developed myelodysplasia and two were considered unfit for any further chemotherapy. Two other patients were scheduled to receive the 3^rd^ consolidation when the charts were reviewed. Therefore the 3^rd^ consolidation cycle was given to 65 patients. No deaths or severe toxicity was noticed after the 3^rd^ consolidation cycle.

### Maintenance

A total of 64 patients received maintenance at a median time of 187 days (range, 89–303) from the start of the induction therapy.^24^,^28^,^49^–^51^ Thirty five patients received ATRA alone (median, 8 cycles), 28 received ATRA+MTX+6-MP (median, 8 cycles) and one patient received only MTX+6-MP because her pseudotumor cerebri symptoms reappeared when ATRA was given. No other grade ≥3 hematologic or non-hematologic toxicity was noticed during maintenance. Fifteen patients are still on maintenance. Four patients discontinued maintenance because of relapse.

## Outcomes

### Early death

The early death rate was 14.9%. (Table 4) Significant predictors of increased risk of early death on univariate analysis were older age, leykocytosis, thrombocytopenia, higher Sanz score, DIC, major hemorrhage, infection at diagnosis and FLT3 ITD mutation (Table 5). No association of early death with morphologic subtype, secondary APL, transcript breakpoint, FLT3 TKD, karyotypic group or gender could be detected. In multivariate logistic regression, baseline characteristics that remained in the final model as independent predictors of early death were DIC, major bleeding and higher age (Table 5).^32^,^33^,^71^

### Response to treatment

**Figure 1:**
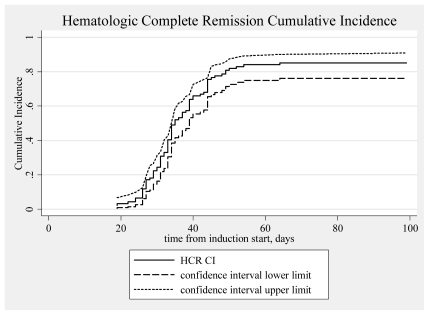
Cumulative incidence of hematologic response (N=94).

**Figure 2:**
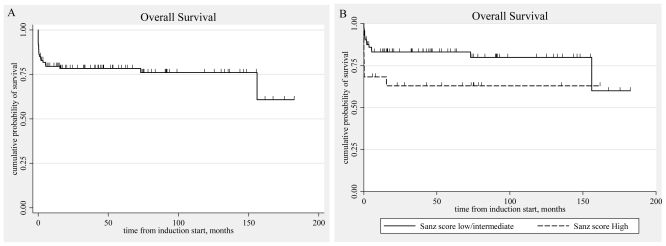
Kaplan-Meier estimated Overall Survival curves. A: all patients (n=94). B: according to Sanz score.

As mentioned above, all 80 patients that completed induction therapy and were evaluated for response achieved CHR. Seven patients died before induction therapy was introduced and eight died early after induction. The cumulative incidence of CHR from the beginning of the induction therapy was 85.1% (95% CI, 76.1–90.9).(Figure 1) At the end of the consolidation therapy 73 of the 75 evaluable patients achieved molecular remission. Only two patients never achieved molecular remission. Both died after aggressive chemotherapy or allogeneic stem cell transplantation.^83^,^90^ With a median follow-up of the surviving patients of 55 months (range, 1.3–182) the OS at 5 years was 78.4% (95% CI, 68.5–85.5). The OS for the patients of the low/intermediate risk group was 83.1% and that of the high risk group 62.9%. (Table 6, Figure 2) If we consider only the patients that received full induction therapy (i.e. until day 8, n=87), the OS at 5 years was 84.7% (95% CI, 75.1–90.8).

**Figure 3:**
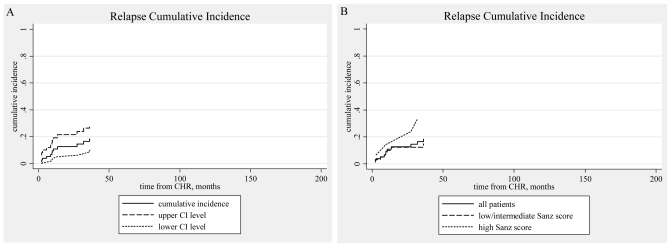
Relapse cumulative incidence. A: all patients (N=80). B: according to Sanz score.

Ten patients relapsed. In seven patients the relapse was molecular and in three hematologic. There was no case of extramedullary relapse. Most of these patients were treated with ATO.^91^,^92^ Two patients died during salvage therapy. Six achieved molecular remission and proceeded to autologous stem cell transplantation. In all these six cases the graft was PML-RARα negative.^91^,^93^ Five of these six patients are alive and in molecular remission for 2–124 months after the transplantation (median, 36). Three relapsed patients did not achieve molecular remission and proceeded to allogeneic stem cell transplantation. All had an HLA identical sibling. They are alive and in molecular remission 62, 70, and 118 months after the transplantation. The cumulative incidence of relapse (calculated from the date of CHR) was 18.3% (95% CI, 9.9–28.7). It was 14.8% for the low/intermediate risk group and 33.5% for the high risk group (univariate, HR=2.21, p=0.195). (Table 6, Figure 3) Two patients developed a myelodysplastic syndrome 4 months and 3 years after the diagnosis of APL.^36^,^37^,^44^,^51^,^94^ Both are alive and in complete molecular remission concerning PML/RARα seven months and eight years since the diagnosis of MDS, respectively.

**Figure 4:**
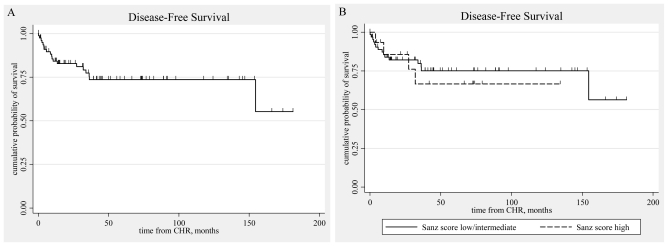
Kaplan-Meier estimated Disease Free Survival curves. A: all patients (n=94). B: according to Sanz score.

The DFS for all 80 patients that achieved CHR was 73.6%. The DFS of the patients belonging to the low/intermediate risk group was 75.1%, and of those belonging to the high risk group 66.5%. (Table 6, Figure 4)

The results of univariate and multivariate analysis concerning overall survival are shown in tables 7 and 8. The same factors were studied with univariate analysis for the DFS and the CIR. None proved to be significant (data not shown).

## Discussion

In this study we present the clinical, immunophenotypic, cytogenetic, and molecular findings as well as the clinical course of 95 Greek APL patients from 6 hospitals in Greece and Cyprus. Since there is not a national registry for this particular type of AML in Greece, the present study represents the largest series ever reported.

Demographics do not seem to differ substantially from previous literature reports.^43^,^45^,^51^ Immunophenotyping is a useful tool for the correct diagnosis. Intermediate to high side scatter, strong CD33 expression and MPO positivity, CD34+/−, CD13++, CD15+ dim and lack of HLA-DR expression were characteristic findings in almost all cases studied. The only patient showing HLA-DR expression was found to be PML-RARα negative since her cytogenetic abnormality was t(11;17).^58^–^61^ We noticed a strong correlation between the expression of lymphoid markers and the development of differentiation syndrome since 7/8 cases expressing lymphoid markers developed the syndrome. By far the most frequent lymphoid marker expressed was CD2, a cell adhesion molecule. An explanation for such a correlation might be the adherence of the cells expressing such molecules to the capillary endothelium with consequent inflammation.^95^ There were only two cases with CD56 expression, a rather low incidence and, therefore, no conclusions about its significance can be drawn. 96

Taking into account that cytogenetically cryptic PML-RARα rearrangements are observed in 4%–6% of APL cases, we found a rather high number of cases with normal cytogenetics, 9/68 (13.2%). Chance and ‘technical reasons’ are the only explanations we can offer for this finding.^5^,^16^,^26^ Additional chromosomal abnormalities were found in 15/59 cases (28.8%) and trisomy 8 was by far the most frequent abnormality. These findings are in accordance to other reports. The presence of additional chromosomal abnormalities did not influence the response to treatment. We therefore agree with the suggestion that ACAs is not a reason to intensify treatment.^11^–^15^ The prognostic significance of ider(17q) and three way translocations of t(15;17) are currently unknown due to their low incidence.^41^ In addition, the third chromosome that is involved in the three way translocation to the t(15;17) varies. One of the two variant translocations involving chromosomes 15, 17 and 18 was associated with a submicroscopic deletion of the 5′ part of the *RARα* gene, as evidenced by FISH.^97^ Because only limited number of studies has examined such deletions associated with the *PML-RARa* fusion gene, their significance and their involvement in the pathogenesis of the disease if any, remain unclear. One of our patients manifested t(8;21) in addition to the t(15;17). Although the patient received induction therapy according to the AIDA protocol and subsequently two cycles of high dose cytarabine with idarubicin and ATRA he did not achieve molecular remission. The co-existence of t(15;17) and t(8;21) in a single leukemic clone is a very rare finding and the results of the few reported cases are conflicting. However, it has been proposed that *PML-RARa* and *AML1/ETO* fusion proteins may mutually affect their pathogenetic mechanism, rendering the cells resistant to ATRA which is in agreement with the resistance of our patient to ATRA based treatment.^38^,^80^,^81^

In the Greek origin patients the L isoform of the PML-RARa was the most frequent and this is in agreement with previous reports. In contrast, in the Cyprus origin patients we noticed a very high frequency of the S isoform.^5^,^17^,^18^ We can offer no explanation for this finding and it definitely needs confirmation. The presence of *FLT3* mutations was associated with various adverse features and generally with intermediate/high risk score, as previously reported. It seems therefore that these mutations are inherently connected with the biology of the disease. We observed one patient showing the *FLT3* TKD permanently for 4 years while being in complete molecular remission. Such a finding supports the suggestion of not using *FLT3* mutations as a marker for minimal residual disease in APL.^22^,^69^,^98^,^99^

One important finding of our study was the considerable early death rate. Actually there were 14/94 early deaths (14.9%), with half of these patients dying within the first few days before they could receive full induction treatment. Hemorrhage was the main cause of early death. (Table 4)^32^,^33^,^35^,^71^ Such a figure is increased compared to the numbers reported in the clinical trials of APL (3%–10%). However, population based studies have confirmed that the early death rate reported in clinical trials does not correspond to real life circumstances and worryingly, such a finding has not appreciably changed in the ATRA era. Delays in referring the patients, delays in establishing diagnosis, and failure to promptly begin specific treatment on clinical suspicion and to provide aggressive supportive therapy are factors explaining such mortality and are the targets of the interventions urgently needed. Moreover, our data suggest that much of the prognostic effect of significant variables on OS outcome largely stems from the effect of these variables on the risk of early death. (Tables 5, 7, and 8) This is also supported by the observation that these variables had no discernible effect on disease-free survival or relapse incidence. As regards patients surviving induction treatment the response was excellent. All 80 evaluable patients achieved complete hematologic remission. At the end of the consolidation therapy 73/75 achieved molecular remission. These results are in accordance to the relevant reports of the protocols that our patients followed (AIDA0493, the LPA1999 and LPA2005 PETHEMA and the European APL1993 and APL2000). 30,35,43–51 Only two patients showed primary resistance, i.e. they never achieved molecular remission. Both died after being treated with aggressive chemotherapy or allogeneic stem cell transplantation. We observed 10 relapses (cumulative incidence of relapse 18.3%, 95% CI 9.9%–28.7%) (Figure 3). Because of the close monitoring most relapses were molecular and therefore the patients were salvaged rather early. The salvage treatment (for the majority ATO and autologous or allogeneic stem cell transplantation) was successful: five patients remain in molecular remission for 2–124 months (median, 36) after autologous stem cell transplantation and three patients for 62–118 months after allogeneic stem cell transplantation. These results further support the importance of the close molecular monitoring after the first line treatment is completed.^65^,^100^–^102^ No CNS relapse was observed in the cohort of our patients in none of whom CNS prophylaxis was given.^27^,^52^ There were two patients that developed myelodysplastic syndrome, an incidence not different from the one reported. 27,36,37,40,44,51,94 We were unable to confirm the value of various well established risk factors for the patients’ outcome. Most probable explanations are the rather small numbers in our study as well as the risk adapted therapy that was provided to most of our patients. In summary, our data confirm the excellent results of the current treatment of the disease, provided that the patient will survive the early period after the disease has been diagnosed.

## Figures and Tables

**Table 1:** Baseline characteristics of patients.

Characteristic	Value

n	95

Median age, y (range)	48 (14–86)

Male, n (%)	39 (41)

APL morphology	
hypergranular n (%)	77 (83)
microgranular n (%)	16 (17)

*De novo,* n (%)	86 (91)
Therapy related, n (%)	9 (9)

WBC (x10^9^/L), median (range)	1.6 (0.3–106.6)

Platelets (x10^9^/L), median (range)	23 (1–275)

Risk category*, n (%)	
low	24 (25)
intermediate	48 (51)
high	23 (24)

* According to Sanz *et al.*

**Table 2:** Therapy related APL. Characteristics of patients.

Gender	Age, y	Primary disease	Treatment	Years between treatment and APL	Morphology	Risk category*	Cytogenetics	PML-RARα isoform	FLT3 mutations
M	65	Bladder carcinoma	Surgical, RT	11	Hypergranular	Intermediate	ND	bcr3	wt
M	25	Hodgkin Lymphoma	Chemo	4	Hypergranular	Low	46XY	bcr1	wt
M	75	Hepatoma	Chemo	3	Hypergranular	High	ND	bcr3	TKD
F	79	Soft tissue sarcoma	Surgical, Chemo, RT	2	Microgranular	Intermediate	46,XX,t(15;17)(q22;q21)	bcr1	ND
F	36	Cervical carcinoma	Surgical, RT	1	Microgranular	Intermediate	46,XX,t(15;17)(q22;q21)	ND	ND
F	67	Breast cancer	Chemo	2	Hypergranular	High	NA	bcr3	ND
M	50	Multiple Sclerosis	Mitoxantrone	22	Hypergranular	High	46,XY,t(15;17)(q22;q21)	bcr1	wt
F	45	Multiple Sclerosis	Mitoxantrone	2	Hypergranular	Intermediate	46XX,add(12q),−17,+mar	bcr1	wt
F	45	Multiple Sclerosis	INF, Mitoxantrone	13	Hypergranular	Intermediate	46XX,inv(9)(p11q13), t(15;17)(q22;q21)	NA	ND

RT: Radiotherapy, ND: not done, NA: not available.

* according to Sanz *et al.*

**Table 3:** Abnormal karyotypes (in 59 from 68 cases studied).

Groups of abnormal Karyotypes	ACA to t(15;17) or CA without t(15;17)	Number of patients
t(15;17)(q22;q21) sole		37
t(15;17)(q22;q21) with ACA*		15
	+8 (sole)/total	3/7
	del(9q)	1
	t(15;17)(q11;q11)	1
	der(17)t(15;17)(q22;q21)	1
	+21,+add(8p)	1
	−Y,+10	1
	+8,+mar	1
	t(8;21),+8,−10	1
	del(Xp),−20,add(21q),+mar	1
	der(15)del(15q)t(15;17)(q22;q21),der(17)t(15;17)(q22;q21)	1
	+8,der(15)t(15;17)(q22;q21),ider(17)(q10),t(15;17)(q22;q21)	1
	t(1;15;17)(q21;q22;q12), der(7)	1
	t(15;18;17)(q22;q21;q21)	1
Variant translocation without the involvement of chromosome 15	t(11;17),−10,der(?)	1
Chromosomal abnormalities without t(15;17) or its variants		6
	8	3
	add(16q)	1
	add(12q),−17,+mar	1
	−15,+19,+mar1,+mar2	1

Abnormal karyotypes categorized into four groups. ACA: additional chromosomal abnormalities, CA: chromosomal abnormalities.

* Patients with der(17)t(15;17), der(15)t(15;17), ider(17q) or three way translocations including chromosomes 15 and 17 were placed in the group of ACAs.

**Table 4:** Early death. Main characteristics of patients.

Characteristic	Distribution
N	14
Day of death, median (range)	6 (1–24) (Day1: 3, Day2: 3, Day4: 1, Day 8: 1, Day 10: 1, Day 12: 1, Day 14: 2, Day 19: 1, Day 24: 1)
Age, median (range)	52.5 (38–84)
Gender	Male 8/14 (57.1%) Female 6/14 (42.9%),
WBC count/uL, median (range)	10795 (850–106600)
PLT count/uL, median (range)	13000 (5000–30000)
Sanz score	Intermediate 7/14 (50%), High 7/14 (50%)
DIC	13/14 (92.9%)
Major hemorrhage	9/14 (64.3%)
Infection	6/12 (50%)
Intracranial bleeding	7/14 (50%)
Number of major bleeding sites	0: 5/14 (35.7%), 1: 6/14 (42.7%), 2: 3/14 (21.4%)
APL variant	Hypergranular: 11/14 (78.6%), Microgranular: 3/14 (21.4%)
Secondary disease	1/14 (7.1%)
PML-RARα isoform	bcr1: 4/12 (33.3%), bcr2: 1/12 (8.3%), bcr3: 7/12 (58.3%)
FLT3	wt: 1/8 (12.5%), ITD: 5/8 (62.5%), TKD: 2/8 (25%)
Karyotype	t(15;17): 3/5 (60%), t(15;17) del 9q: 1/5 (20%), 46XX add 16, 46XX: 1/5 (20%)
Immunophenotype	CD56 negative 5/5, CD7 negative 9/9, CD2 negative 8/8

**Table 5:** Univariate and multivariate logistic regression analysis of early death risk.

Univariate logistic regression			
Variable	OR	p	n
Age (per year increase)	1.053	0.012	94
WBC ≥10000 vs <10000	4.33	0.016	94
PLT <40000 vs ≥40000		0.01 (Pearson χ2)^*^	94
Sanz score high vs low/intermediate	4.33	0.016	94
DIC	12.37	0.018	94
Major hemorrhage	6.67	0.002	94
Infection at diagnosis	2.8	0.104	88
FLT3 ITD	9.72	0.008	49
^*^ None of the pts presenting with PLT≥40000 experienced early death			
**ii. Multivariate logistic regression**			94
**Variable**	**OR**	**p**	
DIC	9.1	0.05	
Major hemorrhage	5.31	0.024	
Age (per year increase)	1.07	0.007	

Variables included in the initial model: age (continuous), high vs low/intermediate Sanz score, DIC major hemorrhage, infection at diagnosis, FLT3 ITD positivity.

**Table 6:** OS, DFS and CIR estimates

Risk group	OS at 5 y (95% CI)	P	DFS at 5 y (95% CI)	P	CIR at 5 y (95% CI)	P
All Patients	78.4% (68.5–85.5)		73.6% (61.0–82.7)		18.3% (9.9–28.7)	
Low/intermediate	83.1% (72.1–90.0)		75.1% (61.2–84.6)		14.8% (6.8–25.7)	
High	62.9% (39.2–79.5)	0.06	66.5% (32.7–86.2)	0.7	33.5% (9.9–59.5)	0.195

**Table 7:** Characteristics and postremission outcome of APL patients (univariate analysis)

		% OS			
Characteristic	No. of patients	at 3 y	at 5 y	HR	p
Overall	94	78.4%	78.4%		
Gender					
Male	56	79.9%	79.9%	0.7	0.35
Female	38	76.2%	76.2%		
Age					
≥60	25	84%	84%	2.92	0.015
<60	69	61%	61%		
WBC					
≥10000	22	62.9%	62.9%	2.25	0.068
<10000	72	83.1%	83.1%		
PLT					
<40000	67	74.4%	74.4%	2.84	0.093
≥40000	27	88.3%	88.3%		
Sanz score					
high	22	62.9%	62.9%	2.25	0.068
low/intermediate	72	83.1%	83.1%		
DIC					
yes	54	68.1%	68.1%	2.72	0.006
no	40	92.4%	92.4%		
MH					
yes	26	57.2%	57.2%	3.46	0.004
no	68	86.5%	86.5%		
FLT3 ITD					
ITD	11	54.6%	54.6%	3.86	0.016
wt/TKD	38	80.7%	80.7%		
FLT3 TKD					
TKD	9	77.8%	77.8%	0.75	0.7
wt/ITD	42	72.8%	72.8%		
Infection at diagnosis					
yes	26	68.6%	68.6%	2	0.136
no	62	83.8%	83.8%		
FAB type					
microgranular	16	81.25%	81.25%	0.77	0.675
hypergranular	76	77.2%	77.2%		
PML-RARα Isoform					
bcr1	38	72.2%	72.2%		
bcr3	33	78.8%	78.8%	0.93	0.88
Karyotype group					
sole t(15;17)	37	83.1%	83.1%		
t(15;17) with ACAs	15	79%	79%	1.26	0.742
Secondary APL					
no	85	72.3%	72.3%		
yes	9	88.9%	88.9%	0.9	0.893

MH: major hemorrhage

**Table 8:** Multivariate Cox regression analysis of Overall Survival

Overall Survival-Multivariate analysis: i. FLT3 ITD included (N=49)		
Variable	HR	p
FLT3 ITD vs wt/TKD	3.48	0.029
Major hemorrhage	3.75	0.028
High vs low Sanz score	2.66	0.093
**Overall Survival-Multivariate analysis: ii. FLT3 ITD not included (N=94)**		
**Variable**	**HR**	**p**
Age (≥60 vs <60)	2.49	0.038
DIC	3.86	0.036
Major hemorrhage	2.22	0.073

Variables included in the initial model: age (≥60 vs <60), Sanz score (high vs low and int vs low or high vs low/int), PLT (<40000 vs ≥40000), DIC, major hemorrhage, infection at diagnosis, FLT3 ITD vs other
